# Deceptive Dyspnea: A Dual Pathology of Myasthenia Gravis and Right-to-Left Shunt Masquerading As Status Asthmaticus

**DOI:** 10.7759/cureus.97413

**Published:** 2025-11-21

**Authors:** Abubakar Gapizov, Ayesha Farooq, Quratulain Jabbar, Muhammad Subhan, Ayesha Khalid, Mehvish Aqil

**Affiliations:** 1 Internal Medicine, Weill Cornell Medicine, New York Presbyterian Brooklyn Methodist Hospital, Brooklyn, USA; 2 Medicine, Liaquat University of Medical and Health Sciences, Jamshoro, PAK; 3 Medicine, Federal Medical and Dental College, Islamabad, PAK; 4 Internal Medicine, Jinnah hospital Lahore, Allama Iqbal Medical College, Lahore, PAK; 5 Cardiology, Shaikh Zayed Hospital, Lahore, PAK; 6 Pulmonology, District Headquarters (DHQ) Hospital/Allied Hospital 2, Faisalabad Medical University, Faisalabad, PAK

**Keywords:** acute hypoxemic respiratory failure, asthma, atrioventricular canal defect, echocardiography - heart failure - valvular heart disease, electrocardiogram (ecg/ekg), idiopathic pulmonary arterial hypertension, intermittent hypoxia, myasthenia gravis (mg), pyridostigmine, refractory status asthmaticus

## Abstract

Respiratory failure in myasthenia gravis (MG) can mimic refractory status asthmaticus, leading to misdiagnosis and delayed treatment. We present the case of a 28-year-old female with a history of asthma and a known partial atrioventricular canal defect who was admitted with acute respiratory failure and severe hypoxemia, not responsive to maximum bronchodilator and corticosteroid therapy. Fatigable weakness on bedside examination led to a diagnosis of seropositive generalized MG. Although treated with pyridostigmine and intravenous immunoglobulin to improve her muscle strength, the persistence of severe hypoxemia suggested an underlying pathology. Bubble contrast echocardiography then confirmed a right-to-left shunt via her cardiac defect, along with mild pulmonary hypertension, and thus a double pathophysiology for her respiratory failure. It highlights that a failure of the patient to improve as anticipated should prompt diagnostic reassessment to prevent anchoring bias, and it underscores the crucial role of contrast echocardiography in identifying disproportionate hypoxemia in patients with an underlying cardiac shunt.

## Introduction

Respiratory failure from isolated neuromuscular weakness can convincingly mimic primary pulmonary disease, leading to critical diagnostic delays [1,2]. Myasthenia gravis (MG), in particular, may present with predominant respiratory muscle involvement, masquerading as refractory status asthmaticus [3]. This misdiagnosis often anchors clinicians to bronchodilators and corticosteroids, postponing life-saving immunotherapy [3]. The diagnostic challenge intensifies when a patient has a comorbidity that independently contributes to hypoxemia [3]. Structural heart disease, such as a partial atrioventricular (AV) canal defect, can lead to intracardiac shunting and secondary pulmonary hypertension [4]. The pathophysiology of hypoxia in such defects is critical to understand, as a partial AV canal defect, comprising a primum atrial septal defect and a cleft mitral valve, typically results in a predominant left-to-right shunt due to higher left-sided cardiac pressures [5]. However, the direction of shunt flow is dynamic, as the detection of microbubbles in the left atrium via a contrast echocardiogram confirms the presence of an anatomic communication permitting right-to-left shunting. Still, it does not, in isolation, quantify its hemodynamic significance at rest [5]. Transient reversal of flow can occur during the cardiac cycle or with maneuvers like Valsalva, even when the net shunt is left-to-right [6]. In the context of acute respiratory failure, factors such as elevated pulmonary vascular resistance and increased right-sided filling pressures can exacerbate this right-to-left flow, transforming a previously well-tolerated defect into a source of profound, refractory hypoxemia [6].

While the misdiagnosis of MG as asthma is documented, the specific scenario where MG and a congenital shunt coexist and synergistically drive respiratory failure is a rarely highlighted clinical phenomenon [5,6]. This convergence represents a significant risk for diagnostic anchoring and demands a systematic approach that investigates multiple organ systems [7]. We present the case of a young woman with a known partial AV canal defect, in whom an initial presentation of status asthmaticus eclipsed the diagnosis of generalized MG. The case underscores the imperative for a dual-pathway evaluation, integrating meticulous neurological examination with a sophisticated understanding of cardiac shunt physiology, in any presentation of unexplained respiratory failure.

## Case presentation

A 28-year-old woman presented to the emergency department with an acute onset of severe, central chest pain radiating to her jaw, accompanied by rapidly progressive dyspnea, orthopnea, and paroxysmal nocturnal dyspnea. Her medical history was significant for insulin-dependent diabetes mellitus, mild hypertension, and a known partial AV canal defect. Notably, she had been hospitalized two months earlier for a severe respiratory event requiring mechanical ventilation. During that prior admission, her presentation with wheezing and rhonchi led to a working diagnosis of "refractory asthma," for which she received standard bronchodilator and ventilator management. The potential that this prior event represented an undiagnosed myasthenic crisis, rather than pure asthma, was only considered in retrospect following her current presentation. No pulmonary function tests had been performed to objectively confirm asthma during the initial hospitalization.

**Figure 1:**
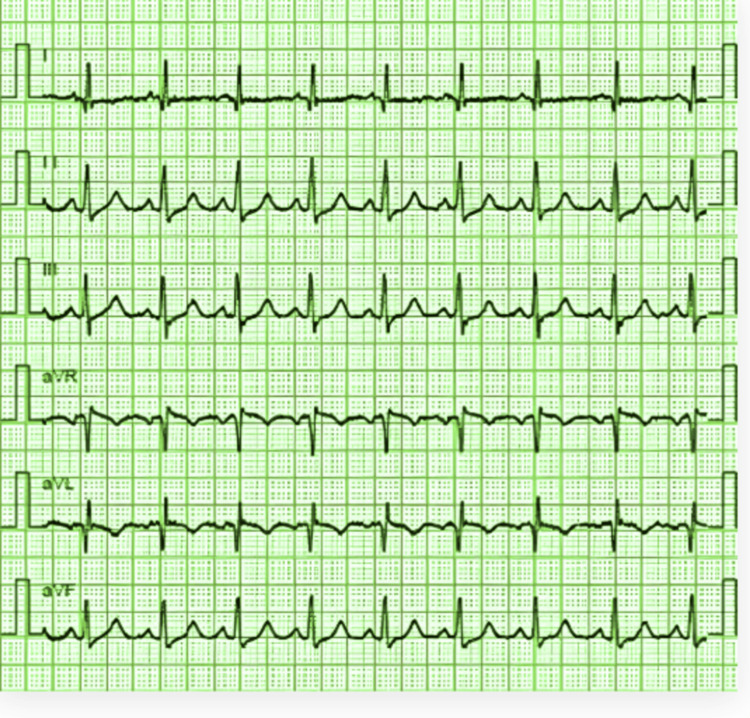
ECG demonstrating sinus tachycardia This ECG, obtained on admission, shows a regular narrow-complex rhythm at a rate of 114 beats per minute. ECG: electrocardiogram

On presentation, she was tachycardic (114 bpm) and hypotensive (90/60 mmHg). She was afebrile but profoundly hypoxic, with an oxygen saturation of 86% on room air. Physical examination revealed decreased bilateral air entry and diffuse rhonchi, initially supporting a working diagnosis of status asthmaticus. However, a careful cardiovascular exam revealed a loud P2 sound, suggestive of pulmonary hypertension. Crucially, a neurological assessment, prompted by her generalized fatigue and difficulty holding her head up, uncovered bilateral ptosis that worsened with sustained upward gaze and rapid arm drooping during maintained abduction, a positive “bat-wing sign” indicative of fatigable weakness. Initial investigations were targeted at common causes of acute dyspnea and chest pain. An ECG showed only sinus tachycardia with no ischemic changes (Figure 1), and serial troponin I levels were negative.

**Figure 2:**
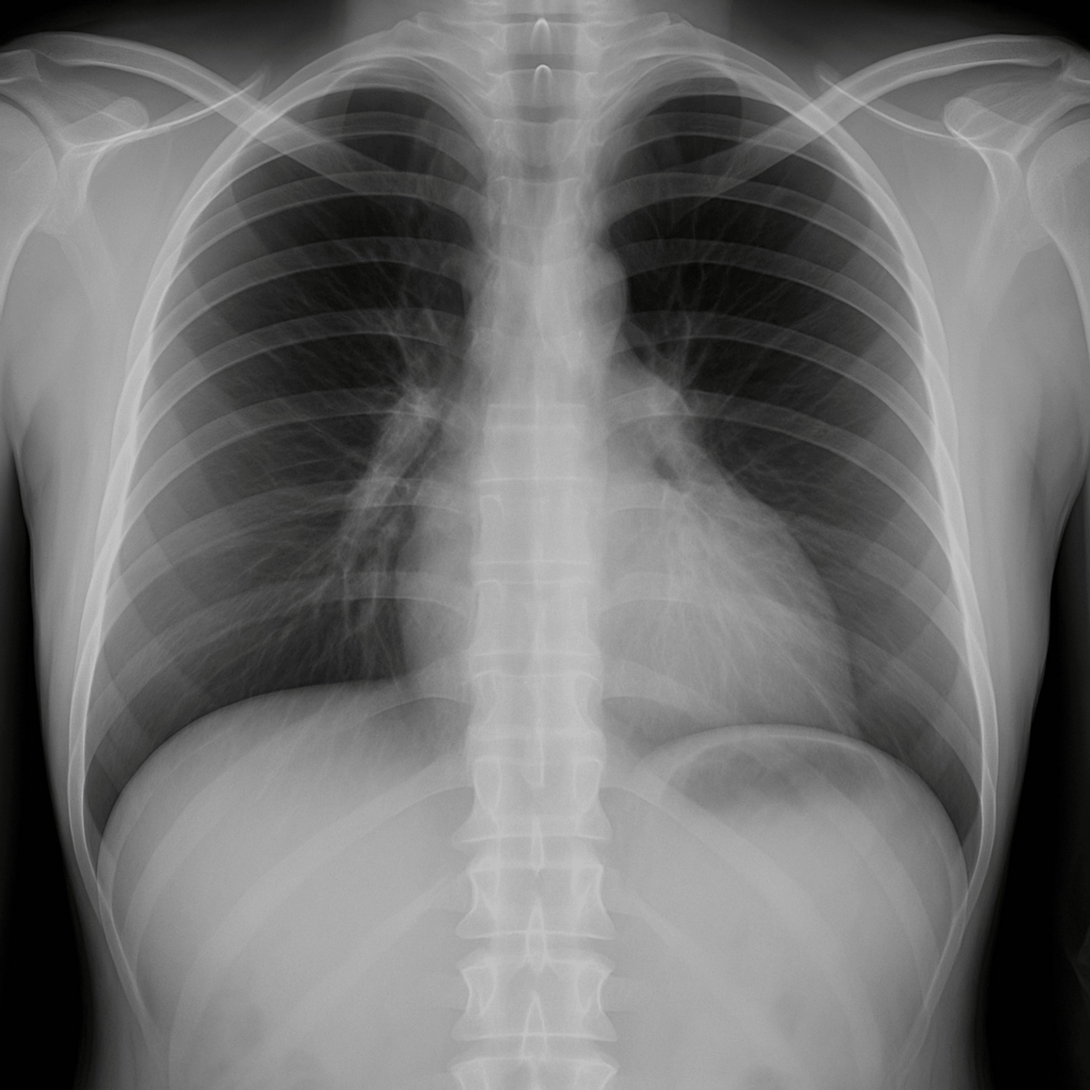
Chest X-ray showing hyperinflated lungs with mild hilar congestion

**Figure 3:**
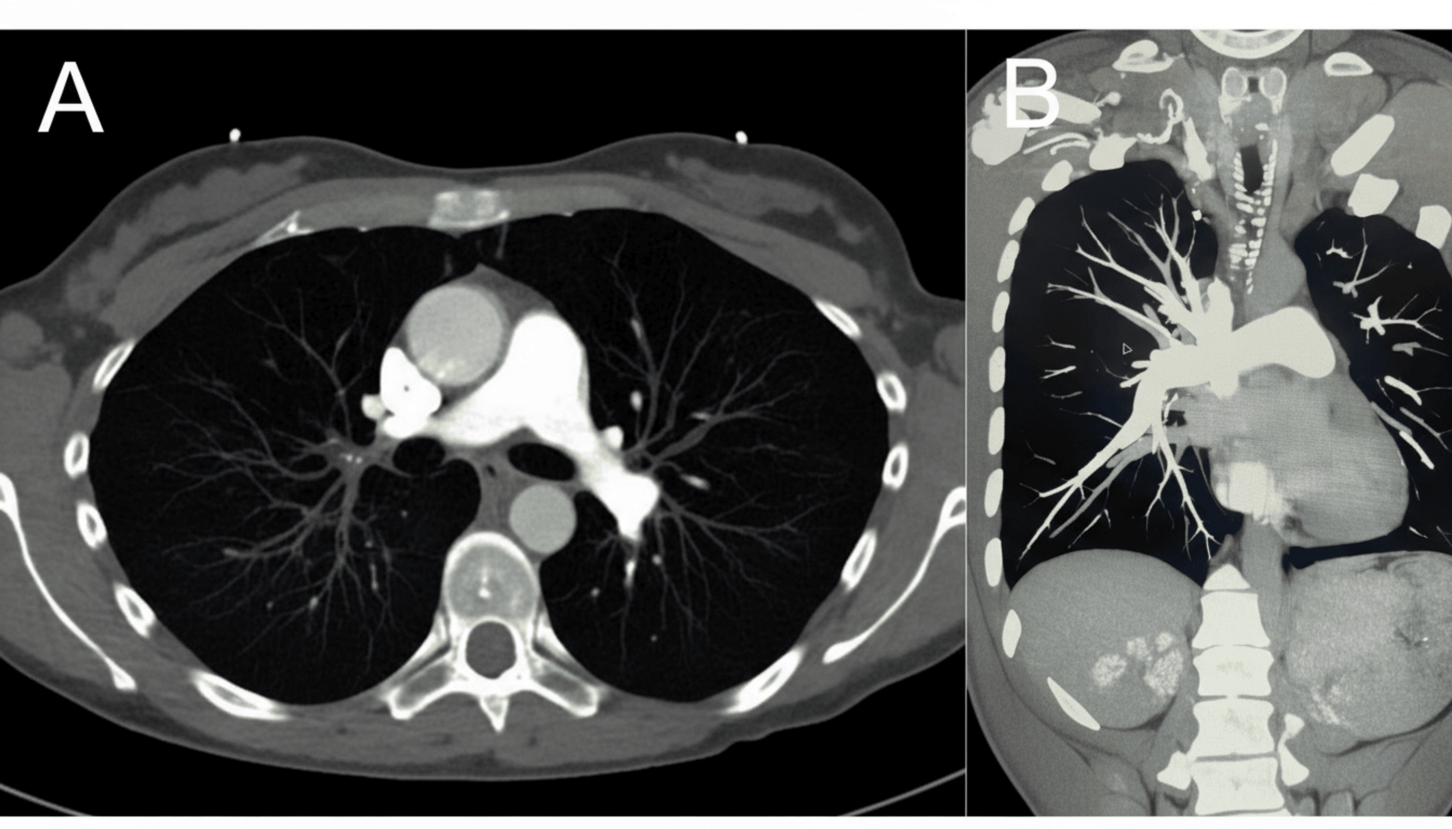
CT angiography of chest (unremarkable) (A) Axial CT pulmonary angiogram showing clear pulmonary arteries with no embolism. (B) Coronal view confirming patent vasculature and absence of interstitial lung disease. CT: computed tomography

A chest radiograph demonstrated hyperinflation without consolidation or pulmonary edema, as shown in Figure 2, and a high-resolution CT angiogram definitively ruled out pulmonary embolism and interstitial lung disease, as shown in Figure 3.

An arterial blood gas on room air revealed profound hypoxemia (PaO₂ 55 mmHg) with borderline hypercapnia (PaCO₂ 45 mmHg), a finding more suggestive of a shunt physiology or severe ventilation-perfusion (V/Q) mismatch than pure airway obstruction. She was managed aggressively for presumed refractory status asthmaticus with high-flow oxygen (FiO₂ 60%), intravenous hydrocortisone, intensive nebulization, and empiric antibiotics. After 48 hours of this maximal therapy, her hypoxemia and respiratory distress showed only mild improvement. Bedside pulmonary function tests showed markedly reduced vital capacity and a weak cough, while maximum inspiratory and expiratory pressures were severely diminished, objectively quantifying respiratory muscle weakness. This objective evidence of neuromuscular impairment, combined with the therapeutic impasse, was the critical turning point that forced a diagnostic reconsideration. A formal bedside neurological assessment was performed, revealing a positive ice pack test. This finding triggered a definitive neuromuscular workup, which showed a >15% decrement on repetitive nerve stimulation and strongly positive anti-acetylcholine receptor (AChR) antibody testing, confirming generalized MG. Table 1 summarizes the patient’s initial routine blood investigations and specialized serological testing. The results are notable for severe hypoxemia and hypercapnia on arterial blood gas, with an otherwise largely unremarkable metabolic, infectious, and inflammatory profile. The elevated blood glucose and HbA1c reflect the patient’s known, suboptimally controlled insulin-dependent diabetes mellitus.

**Table 1:** Comprehensive laboratory profile at initial presentation Each test category represents a grouped section of related investigations. ALT: alanine aminotransferase, AST: aspartate aminotransferase, AChR: acetylcholine receptor, BNP: B-type natriuretic peptide, BUN: blood urea nitrogen, CK-MB: creatine kinase-myocardial band, CRP: C-reactive protein, DM: diabetes mellitus, ESR: erythrocyte sedimentation rate, HbA1c: hemoglobin A1c, HCO₃: bicarbonate, MI: myocardial infarction, MuSK: muscle-specific kinase, PaCO₂: partial pressure of carbon dioxide (arterial), PaO₂: partial pressure of oxygen (arterial), TSH: thyroid-stimulating hormone, T4: thyroxine, WBC: white blood cell count, MG: myasthenia gravis

Specific test	Patient value	Normal range	Interpretation
Arterial blood gas			
pH	7.36	7.35-7.45	Normal acid-base status
PaO₂	55 mmHg	80-100 mmHg	Severe hypoxemia
PaCO₂	45 mmHg	35-45 mmHg	Borderline hypercapnia
HCO₃	24 mEq/L	22-26 mEq/L	Normal bicarbonate
Oxygen saturation	86%	95-100%	Severe desaturation
Cardiac markers			
Troponin I	0.01 ng/mL	<0.04 ng/mL	Normal (ruled out MI)
CK-MB	4 ng/mL	0-5 ng/mL	Normal
BNP	85 pg/mL	<100 pg/mL	Normal
Complete blood count			
Hemoglobin	12.8 g/dL	12.0-15.5 g/dL	Normal
Hematocrit	38%	36-48%	Normal
WBC count	9.2 × 10³/μL	4.5-11.0 × 10³/μL	Normal
Neutrophils	68%	40-70%	Normal
Lymphocytes	25%	20-40%	Normal
Platelets	245 × 10³/μL	150-450 × 10³/μL	Normal
Inflammatory markers			
ESR	18 mm/hr	0-20 mm/hr	Normal
CRP	0.8 mg/dL	<1.0 mg/dL	Normal
Procalcitonin	0.05 ng/mL	<0.1 ng/mL	Normal
Liver function tests			
ALT	28 U/L	7-45 U/L	Normal
AST	32 U/L	10-35 U/L	Normal
Alkaline phosphatase	85 U/L	45-115 U/L	Normal
Total bilirubin	0.8 mg/dL	0.1-1.2 mg/dL	Normal
Albumin	3.9 g/dL	3.5-5.0 g/dL	Normal
Renal function and electrolytes			
Sodium	138 mEq/L	136-145 mEq/L	Normal
Potassium	4.1 mEq/L	3.5-5.1 mEq/L	Normal
Chloride	102 mEq/L	98-107 mEq/L	Normal
Bicarbonate	25 mEq/L	22-28 mEq/L	Normal
BUN	18 mg/dL	7-20 mg/dL	Normal
Creatinine	0.9 mg/dL	0.6-1.1 mg/dL	Normal
Glucose	245 mg/dL	70-110 mg/dL	Elevated (known DM)
Thyroid function			
TSH	2.1 mIU/L	0.4-4.0 mIU/L	Normal
Free T4	1.1 ng/dL	0.8-1.8 ng/dL	Normal
MG panel			
AChR binding antibodies	8.5 nmol/L	<0.4 nmol/L	Positive
AChR modulating antibodies	92%	<25%	Positive
MuSK antibodies	Negative	Negative	Negative
Other relevant tests			
HbA1c	8.2%	<6.5%	Elevated (poor control)
Lactic acid	1.8 mmol/L	0.5-2.2 mmol/L	Normal
Magnesium	2.1 mg/dL	1.8-2.4 mg/dL	Normal
Calcium	9.1 mg/dL	8.5-10.5 mg/dL	Normal

**Figure 4:**
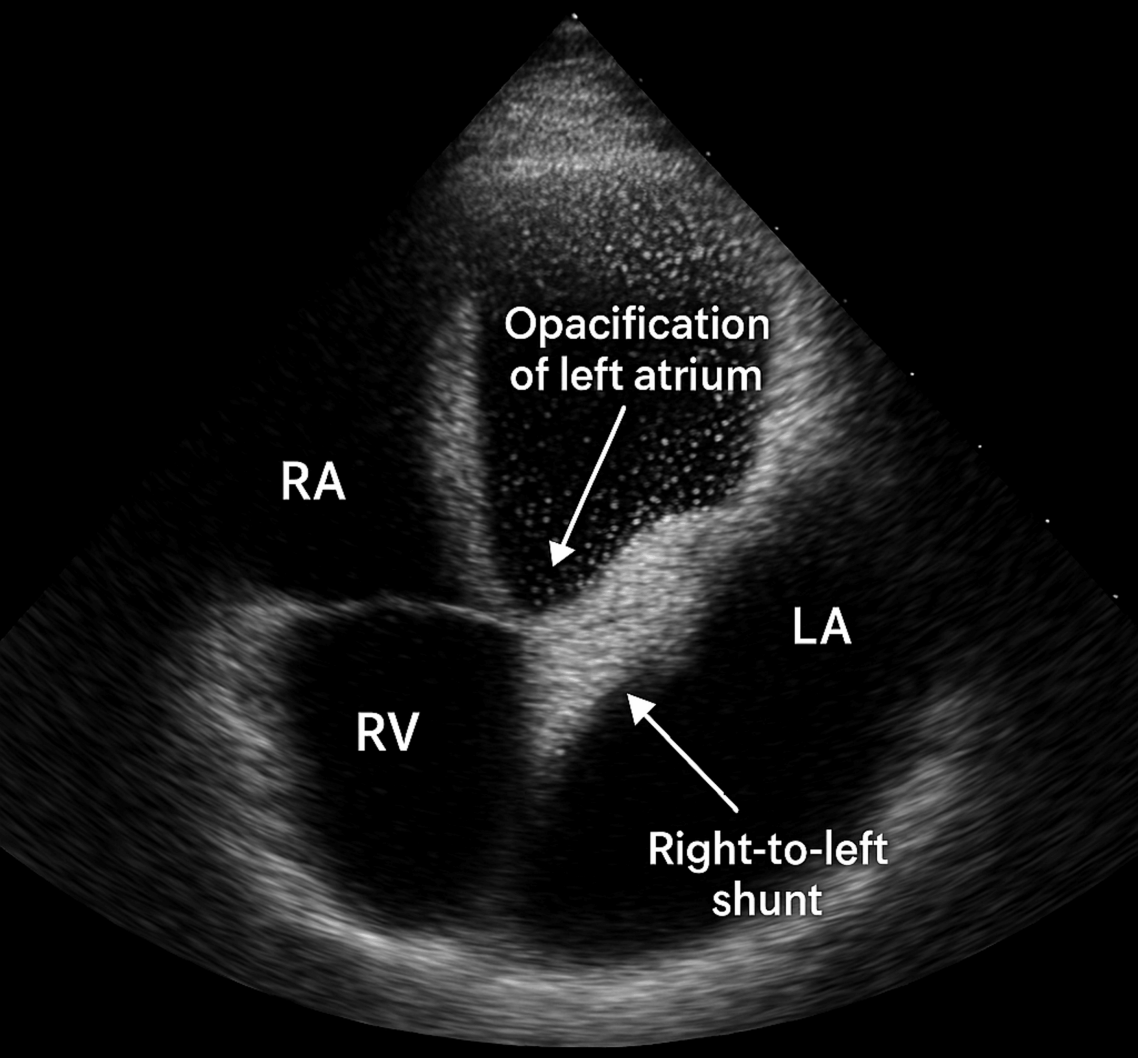
Transthoracic echocardiography showing opacification of LA and right to left shunt RA: right atrium, RV: right ventricle, LA: left atrium

Treatment for MG was initiated immediately with intravenous immunoglobulin (IVIG, 2 g/kg over five days) and pyridostigmine (60 mg every six hours). This was followed by oral prednisolone (1 mg/kg daily) and azathioprine for long-term immunosuppression after normal thiopurine methyltransferase levels were confirmed. While her muscle strength and fatigability improved dramatically over the subsequent week, a significant oxygen requirement persisted, with her PaO₂ remaining around 60 mmHg on 4 L/min of oxygen via nasal cannula. This discordance-improving strength but persistent hypoxemia prompted a re-evaluation of her known cardiac defect. Figure 4 shows the echocardiographic features of right-to-left shunt and opacification of the left atrium.

**Figure 5:**
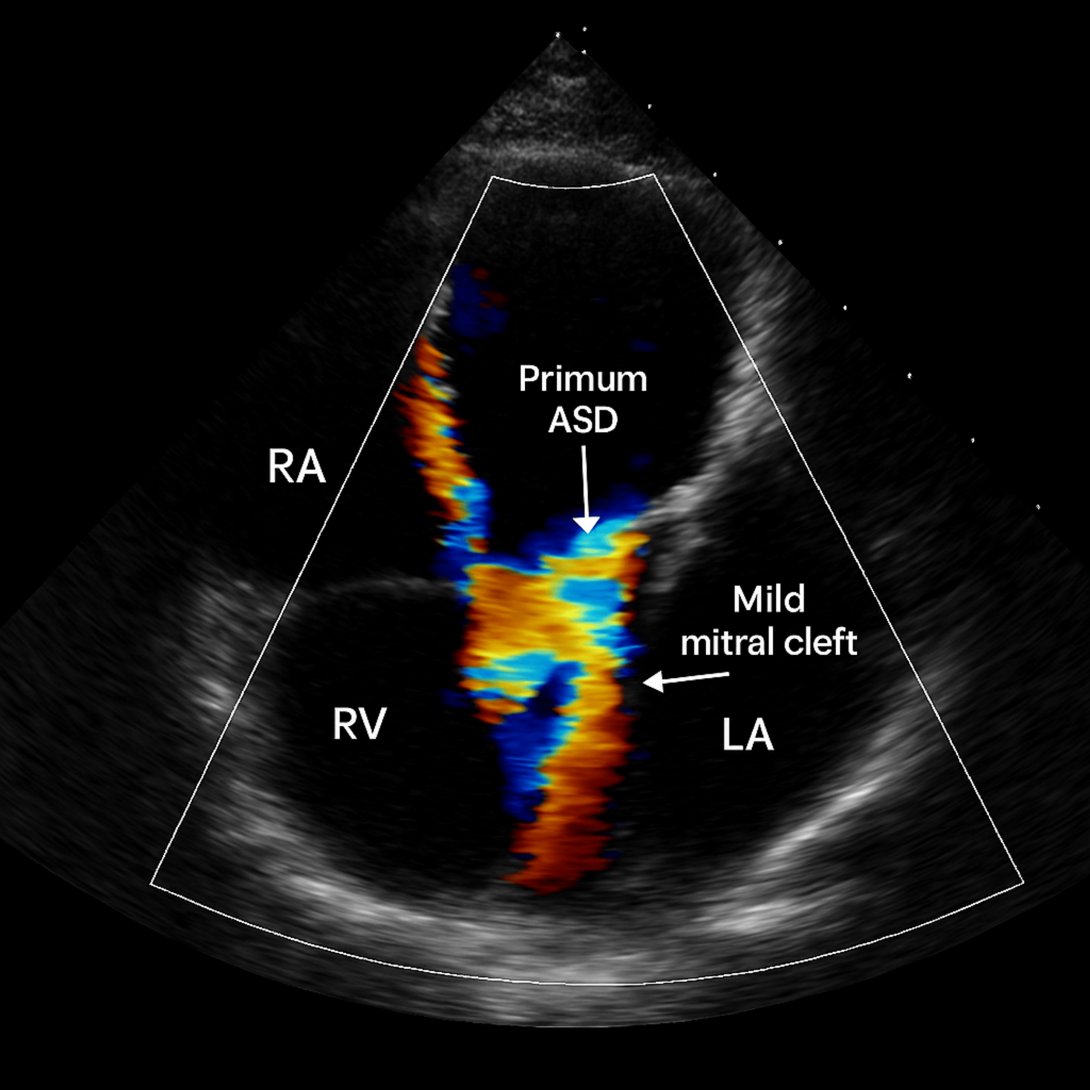
Bubble contrast echocardiography revealed early opacification of the left atrium, mild mitral cleft and primum ASD RA: right atrium, RV: right ventricle, LA: left atrium, ASD: atrial septal defect

A repeat transthoracic echocardiogram with an agitated saline bubble study was performed. It confirmed the partial AV canal defect with moderate post-capillary pulmonary hypertension (estimated PASP of 45 mmHg) secondary to the chronic left-to-right shunt, as quantified by a shunt fraction (Qp/Qs) of 1.5:1. Critically, the bubble study revealed early opacification of the left atrium within three cardiac cycles, confirming an anatomic right-to-left shunt (Figure 5). This finding resolves the apparent paradox of profound hypoxemia in the setting of a net left-to-right shunt: the fixed defect permitted small-volume right-to-left shunting. While this shunted volume was hemodynamically insignificant under normal conditions, it became critically crucial during her myasthenic crisis. The shunting of severely desaturated blood (PaO₂ 55 mmHg) from the hypoventilated lungs directly into the systemic circulation acted as a significant anatomic shunt, accounting for the refractory nature of her hypoxemia despite high-flow oxygen. This combination of a fixed cardiac defect and acute neuromuscular failure created a unique physiological scenario for compounded respiratory compromise.

A multidisciplinary team comprising neurology, cardiology, and cardiothoracic surgery recommended a conservative cardiac management plan. This included oral furosemide 20 mg daily for volume management to reduce left atrial pressure and potentially lessen the shunt, with close outpatient echocardiographic monitoring. Initiation of pulmonary vasodilators, such as sildenafil, was deferred pending neurological stabilization and reassessment. On this combined regimen, her oxygen requirements progressively declined, and she was successfully weaned to room air within two weeks of admission. She was discharged on pyridostigmine, a tapering dose of prednisolone, azathioprine, and furosemide, with structured follow-up plans in both neurology and adult congenital heart disease clinics to monitor her MG and determine the future need for cardiac surgical intervention.

## Discussion

This case demonstrates how dual, concurrent pathologies can present a diagnostic and therapeutic dilemma in acute respiratory failure. The initial presentation mimicked refractory status asthmaticus, an acknowledged pitfall in MG. There have been reports of MG presenting as severe asthma, with consequential delay in diagnosis and unwarranted intubation [1,2]. Gilhus et al. emphasized that isolated respiratory muscle weakness without overt bulbar or ocular signs is a key contributor to misdiagnosis [1]. While previous reports, such as those by Toshniwal et al. and Shahul et al., have rightly emphasized the potential for MG to mimic refractory asthma, our case extends this clinical lesson into a more complex, multi-systemic domain [2,3]. A comprehensive literature review revealed no prior reported cases of myasthenic crisis occurring in conjunction with a congenital partial AV canal defect leading to significant right-to-left shunting. The pivotal distinction in our patient was the discordant response to immunotherapy: improved muscle strength without resolution of profound hypoxemia. This forced a secondary investigation that identified a significant right-to-left cardiac shunt, a compounding pathology rarely documented in conjunction with a myasthenic crisis. Consequently, this case moves beyond the established narrative of a single diagnostic pitfall to illustrate a novel paradigm of dual pathology, in which the failure to improve after appropriate treatment for one condition must actively prompt the search for another [2,3].

However, our case is distinguished by the critical complicating factor of a congenital cardiac shunt. Whereas the occurrence of MG as an asthmatic mimic has been reported, the particular association of MG with a partial AV canal defect leading to massive right-to-left shunting is rare in the literature. The direct analogy search is negative in published cases, representing a new finding identifying a previously underemphasized diagnostic interaction [3,4]. It is essential to understand the pathophysiological interaction in this case, as MG primarily affects hypoventilation and hypercapnia through fatigue of the respiratory muscles [4]. Although this may precipitate mild hypoxemia, the fixed right-to-left shunt, as detected by bubble echocardiography, accounted for the severe and out-of-proportion hypoxemia (PaO₂ 55 mmHg) unresponsive to high-flow oxygen [5]. The shunt creates a true intrapulmonary ventilation-perfusion (V/Q) mismatch that is not corrected by improving minute ventilation alone [5]. This synergy, in which MG limits ventilation and the shunt bypasses the pulmonary capillary bed, creates compounded respiratory failure inadequately addressed by targeting either pathology in isolation [5,6]. The initial clinical improvement in muscle strength with IVIG and pyridostigmine, without concomitant resolution of hypoxia, was the key clue that prompted a search for a second mechanism [7,8]. This sequence highlights a salient learning point: an incomplete clinical response following the appropriate treatment of an initial diagnosis must prompt an immediate search for comorbid or alternative explanations [9].

From an organizational perspective, this case requires a practical, multidisciplinary approach [9]. Optimal care necessitated neurologists controlling immunosuppression, cardiologists tracking shunt hemodynamics and pulmonary pressures, and intensivists optimizing respiratory support [10]. One specific challenge was maximizing preload: diuretics were weighed to cut shunt flow and pulmonary edema. However, their use was tempered by the need to avoid hypovolemia or electrolyte shifts that could exacerbate myasthenic weakness [10]. This scenario is a classic example of cognitive bias in medicine, specifically anchoring heuristics. The patient’s prior asthma history and wheezing on exam anchored the team to a pulmonary obstructive model, delaying the consideration of neuromuscular and cardiac etiologies [7]. The most significant clinical lessons from this case are multifaceted.

Firstly, the presence of unexplained or disproportionate hypoxemia in a patient with presumed asthma must immediately elicit a bedside evaluation for fatigable weakness. Simple, rapid maneuvers, such as the ice pack test for ptosis and prolonged arm-abduction assessment, can provide critical clues pointing to a neuromuscular etiology. Secondly, in any patient with a known congenital heart defect who presents with intractable hypoxia, contrast echocardiography (a bubble study) is an essential diagnostic tool to determine the magnitude and clinical significance of an intracardiac shunt. Finally, a discordant clinical response, such as improvement in muscular strength without the parallel resolution of hypoxemia following appropriate therapy, should be recognized as a significant red flag, indicating a multifactorial process that demands a broader diagnostic investigation beyond the initial diagnosis [9].

We acknowledge the limitations inherent in a single case report. We cannot establish causality or prevalence, and the long-term outcome of managing this dual pathology conservatively (without cardiac surgical intervention) remains to be seen. A specific diagnostic limitation was the inability to precisely quantify the relative contributions of the shunt and hypoventilation to the patient’s hypoxia at presentation [10]. Future directions for research could include systematic screening for cardiac shunts in MG patients with disproportionate hypoxia, or vice versa, screening for neuromuscular disorders in adults with congenital heart disease and unexplained respiratory symptoms [10]. This article adds a new twist to the literature by outlining the intricate interplay between MG with predominant respiratory involvement and shunt physiology. It reaffirms that refractory respiratory failure requires a systematic, multi-system workup that actively seeks out and encompasses multiple possible etiologies.

## Conclusions

This case highlights that refractory respiratory failure requires concomitant assessment of multiple etiologies and against anchoring on a single diagnosis. The key finding was a discordant clinical course: enhanced muscle strength with immunotherapy but no normalization of hypoxia, which led to the identification of an essential cardiac shunt. We recommend a two-pathway strategy in such a situation: standard bedside testing for fatigable weakness and low-suspicion bubble-contrast echocardiography if hypoxemia is disproportionate. This approach is necessary to counter diagnostic anchoring, avoid managerial mistakes, and explicitly confront the multifactorial pathology of intricate respiratory failure.

## References

[1] Gilhus NE, Tzartos S, Evoli A, Palace J, Burns TM, Verschuuren JJ (2019). Myasthenia gravis. Nat Rev Dis Primers.

[2] Toshniwal S, Wanjari A, Acharya S, Kumar S, Sontakke T (2024). Myasthenia gravis mimicking status asthmaticus: the hidden crisis. Cureus.

[3] Shahul HA, Manu MK, Mohapatra AK (2021). Steroid dependence in acute asthma due to myasthenia gravis. Lung India.

[4] Baumgartner H, De Backer J, Babu-Narayan SV (2021). 2020 ESC Guidelines for the management of adult congenital heart disease. Eur Heart J.

[5] Muppidi S, Silvestri NJ, Tan R, Riggs K, Leighton T, Phillips GA (2022). Utilization of MG-ADL in myasthenia gravis clinical research and care. Muscle Nerve.

[6] Stout KK, Daniels CJ, Aboulhosn JA (2019). 2018 AHA/ACC guideline for the management of adults with congenital heart disease: a report of the American College of Cardiology/American Heart Association Task Force on clinical practice guidelines. Circulation.

[7] Warnes CA (2017). Adult congenital heart disease: the challenges of a lifetime. Eur Heart J.

[8] Goldstein SA, Krasuski RA (2022). Pulmonary hypertension in adults with congenital heart disease. Cardiol Clin.

[9] Zulueta JJ, Fanburg BL (1994). Respiratory dysfunction in myasthenia gravis. Clin Chest Med.

[10] Kocabas ZU, Kizilay F, Basarici I, Uysal H (2018). Evaluation of cardiac autonomic functions in myasthenia gravis. Neurol Res.

